# Differentiation of malignant tumours from granulomas by using dynamic [^18^F]-fluoro-L-α-methyltyrosine positron emission tomography

**DOI:** 10.1186/s13550-015-0109-z

**Published:** 2015-04-30

**Authors:** Aiko Yamaguchi, Hirofumi Hanaoka, Yutaka Fujisawa, Songji Zhao, Kazutomo Suzue, Akihiro Morita, Hideyuki Tominaga, Tetsuya Higuchi, Hajime Hisaeda, Yoshito Tsushima, Yuji Kuge, Yasuhiko Iida

**Affiliations:** Department of Bioimaging Information Analysis, Gunma University Graduate School of Medicine, 3-39-22 Showa-machi, Maebashi, Japan; Faculty of Pharmaceutical Sciences, Suzuka University of Medical Science, 3500-3 Minamitamagaki-Cho, Suzuka, Mie 510-8760 Japan; Department of Tracer Kinetics & Bioanalysis, Graduate School of Medicine, Hokkaido University, 5 Chome Kita 8 Jōnishi, Sapporo, Japan; Department of Molecular Imaging, Graduate School of Medicine, Hokkaido University, 5 Chome Kita 8 Jōnishi, Sapporo, Japan; Department of Parasitology, Gunma University Graduate School of Medicine, 3-39-22 Showa-machi, Maebashi, Japan; Advanced Clinical Research Center, Fukushima Medical University, 1 Hikariga-oka, Fukushima, Japan; Department of Diagnostic Radiology and Nuclear Medicine, Gunma University Graduate School of Medicine, 3-39-22 Showa-machi, Maebashi, Japan; Department of Integrated Molecular Imaging, Graduate School of Medicine, Hokkaido University, 5 Chome Kita 8 Jōnishi, Sapporo, Japan; Central Institute of Isotope Science, Hokkaido University, 5 Chome Kita 8 Jōnishi, Sapporo, Japan

**Keywords:** 3-[^18^F]-Fluoro-α-methyl-L-tyrosine, Granuloma, Inflammation, Tumour, Dynamic positron emission tomography

## Abstract

**Background:**

Previous clinical studies have revealed the potential of [^18^F]-fluoro-L-α-methyltyrosine (^18^F-FAMT) for the differential diagnosis of malignant tumours from sarcoidosis. However, one concern regarding the differential diagnosis with ^18^F-FAMT is the possibility of false negatives given the small absolute uptake of ^18^F-FAMT that has been observed in some malignant tumours. The aim of this study was to evaluate a usefulness of dynamic ^18^F-FAMT positron emission tomography (PET) for differentiating malignant tumours from granulomas.

**Methods:**

Rats bearing both granulomas (*Mycobacterium bovis bacillus Calmette-Guérin* (BCG)-induced) and tumours (C6 glioma cell-induced) underwent dynamic 2-deoxy-2-[^18^F]-fluoro-D-glucose (^18^F-FDG) PET and ^18^F-FAMT PET for 120 min on consecutive days. Time-activity curves, static images, mean standardized uptake values (SUVs) and the SUV ratios (SUVRs; calculated by dividing SUV at each time point by that of 2 min after injection) were assessed.

**Results:**

In tumours, ^18^F-FAMT showed a shoulder peak immediately after the initial distribution followed by gradual clearance compared with granulomas. Although the mean SUV in the tumours (1.00 ± 0.10) was significantly higher than that in the granulomas (0.88 ± 0.12), a large overlap was observed. In contrast, the SUVR was markedly higher in tumours than in granulomas (50 min/2 min, 0.72 ± 0.06 and 0.56 ± 0.05, respectively) with no overlap. The dynamic patterns, SUVR, and mean SUV of ^18^F-FDG in the granulomas were comparable to those in the tumours.

**Conclusions:**

Dynamic ^18^F-FAMT and SUVR analysis might compensate for the current limitations and help in improving the diagnostic accuracy of ^18^F-FAMT.

## Background

The differential diagnosis of benign from malignant lesions by using positron emission tomography (PET) is a problem that remains unsolved because 2-deoxy-2-[^18^F]-fluoro-D-glucose (^18^F-FDG) - the most frequently applied PET probe for tumour imaging - shows increased uptake in several benign pathologies such as inflammatory foci, sarcoidosis, and active tuberculosis [1,2]. To overcome this limitation, one promising approach is the use of ^11^C/^18^F-labelled amino acid PET tracers. In contrast to ^18^F-FDG, amino acid tracers show lower uptake in macrophages and other inflammatory cells [3] and thus have been evaluated for their potential to differentiate between benign and malignant lesions, both in animal models and in patients [4-6]. However, several studies have reported increased uptake of amino acid tracers in inflammatory lesions including radiation-induced necrosis [7] and lymphadenopathy in sarcoidosis [8]. Therefore, the use of amino acid tracers for the differential diagnosis has yet to reach a consensus.

In contrast to other amino acid PET tracers, [^18^F]-fluoro-L-α-methyltyrosine (^18^F-FAMT) is selective for L-type amino acid transporter 1 (LAT-1) and interacts less with other transporters [9]. In a previous study that evaluated sarcoidosis patients with suspected malignancy, ^18^F-FAMT showed significantly lower uptake in sarcoidosis lesions compared to tumour lesions, supporting the clinical utility of ^18^F-FAMT for the differential diagnosis [10]. However, one concern regarding the differential diagnosis with ^18^F-FAMT is the possibility of false negative results, because ^18^F-FAMT shows relatively small absolute uptake, even in malignant tumours, which is occasionally lower than that in sarcoidosis. Therefore, an alternative parameter that can estimate differences in ^18^F-FAMT uptake between inflammation and tumours is needed.

Recently, Zhao et al. [11] reported the usefulness of dynamic PET imaging of L-^11^C-methionine (^11^C-MET; the most frequently used amino acid tracer) for the differential diagnosis of granulomas from malignant tumours, in rat models. This tracer exhibited a significantly different dynamic profile in granulomas compared to tumours. The success of dynamic PET using ^11^C-MET for the differential diagnosis raised the intriguing possibility that ^18^F-FAMT could be used as an alternative amino acid PET tracer. However, the dynamic profile of ^18^F-FAMT was unknown, and several characteristics of ^18^F-FAMT, such as the mechanism of transport and metabolism, could lead to a dynamic profile distinct from that of ^11^C-MET. Therefore, in this study, we evaluated the usefulness of dynamic ^18^F-FAMT PET for the differentiation of malignant tumours from granulomas in comparison with ^18^F-FDG in an experimental rat model.

## Methods

### Radiopharmaceuticals

The synthesis of ^18^F-FAMT was performed at the Gunma University Hospital (Cyclotron Facility) as previously described [12]. The radiochemical yield and purity of ^18^F-FAMT were approximately 20% and 99%, respectively. The ^18^F-FDG was also produced in the Cyclotron Facility as previously described [13].

### Animal model

All experimental protocols were approved by the Laboratory Animal Care and Use Committee of Gunma University. Seven-week-old male Wistar King Aptekman/hok rats (weight, 240 to 290 g; Japan SLC, Inc., Shizuoka, Japan) were used in all experiments. We adopted the *Mycobacterium bovis bacillus Calmette-Guérin* (BCG)-induced intramuscular granuloma model that has a similar histological feature as sarcoidosis [14]. The BCG strain of Japan (1 × 10^7^ colony-forming unit (CFU); Japan BCG Laboratory, Tokyo, Japan) was suspended in 0.2 ml of phosphate-buffered saline [15], or allogenic rat glioma cells (C6, 2 × 10^6^ cells/0.2 ml) were inoculated into the left and right calf muscles of the animals to generate a rat model bearing both granulomas and tumours. The C6 glioma cell line (RCB2854) was provided by the RIKEN BRC through the National BioResource Project of the MEXT, Japan.

### Dynamic PET study

At 20 days after the inoculation of BCG and 10 days after the inoculation of the glioma cells, rats (*n* = 6) were kept fasting overnight, anaesthetized with isoflurane, and injected intravenously with ^18^F-FDG (11.7 ± 2.57 MBq). Dynamic PET (list mode acquisition) was performed for 120 min with the hind leg region in the field of view by using an Inveon PET scanner dedicated to small animal imaging (Siemens Medical Solutions USA Inc., Knoxville, TN, USA). The next day, ^18^F-FAMT (19.4 ± 1.91 MBq) was injected intravenously, and dynamic PET was performed according to a similar protocol. The injection doses of ^18^F-FDG and ^18^F-FAMT were determined based on the half-life of ^18^F (110 min) [11] and the tumour accumulation levels of ^18^F-FDG and ^18^F-FAMT.

The data were reconstructed and corrected for attenuation and scatter by using two-dimensional filtered backprojection. The image matrix was 128 × 128 × 159, which resulted in a voxel size of 0.77 × 0.77 × 0.796 mm. The individual time sequence was the following: 4 scans × 1 min, 3 scans × 2 min, 4 scans × 5 min and 9 scans × 10 min. For quantitative analysis, images were analysed by manually drawing volumes of interest (VOIs) in order to trace the contours of the granulomas and tumours without correction for partial volume effects. The time-activity curves, static images (50 to 60 min for ^18^F-FAMT and ^18^F-FDG) and the mean SUVs in the lesions were calculated. The SUV was determined by using the following equation: SUV = activity in a VOI (MBq/cc)/[injected dose (MBq)/body weight (g)].

To further evaluate the dynamic pattern of ^18^F-FAMT uptake, the SUV ratio (SUVR) was determined. The ratio of SUVs of ^18^F-FAMT at each time point to that at 2 min post injection (p.i.) was calculated. The SUVRs of ^18^F-FDG were also calculated from the SUVs at 120 min p.i. relative to that of 2 min p.i.

### Immunofluorescent histochemistry

Paraformaldehyde-fixed (4%), paraffin-embedded, 3-μm-thick tumour and granuloma tissue sections were stained with haematoxylin and eosin (H&E). Immunofluorescent staining of the adjacent tissue sections was also performed according to a standard protocol. Glucose transporter 1 (GLUT-1) was detected by using an anti-GLUT-1 polyclonal antibody (rabbit IgG, synthetic peptide, Abcam, Cambridge, UK). LAT-1 was stained by using an anti-LAT-1 mAb (rabbit IgG, synthetic peptide, Abcam). The primary antibodies were detected with the corresponding isotype-specific goat anti-rabbit IgG H&L (DyLight 488, Abcam). Adjacent tissue sections incubated with rabbit IgG instead of the primary antibodies were used as negative controls.

### Statistical analyses

Repeated measures analysis of variance was used to assess the significance of differences in the dynamic patterns of ^18^F-FAMT and ^18^F-FDG uptake between granulomas and tumours. A paired *t*-test was performed to evaluate the significance of differences in SUVs and SUVRs. A two-tailed value of *p* < 0.05 was considered significant.

## Results

### Dynamic PET study

**Figure 1:**
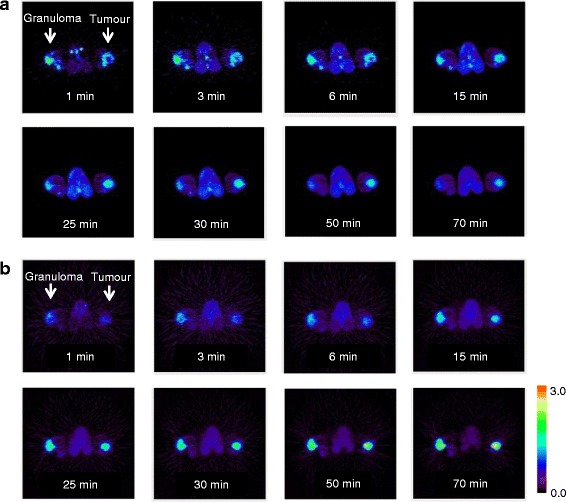
Dynamic ^18^F-FAMT **(a)** and ^18^F-FDG **(b)** images in rats bearing both granuloma and tumour. White arrow: locations of C6 tumour (right) and granuloma (left).

**Figure 2:**
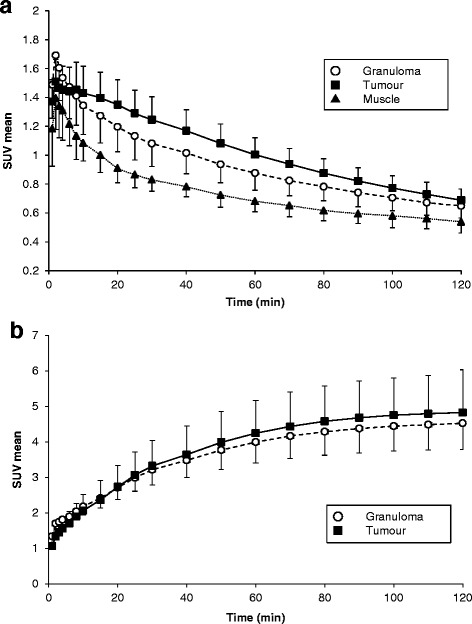
Time-activity curves of ^18^F-FAMT **(a)** and ^18^F-FDG **(b)** in rats bearing tumour and granuloma.

Representative transversal dynamic images of ^18^F-FAMT and ^18^F-FDG are shown in Figure 1. The uptake of ^18^F-FAMT gradually decreased in both granulomas and tumours (Figure 1a). In contrast, the uptake of ^18^F-FDG gradually increased in both lesions (Figure 1b). The time-activity curves of the mean SUVs of ^18^F-FAMT and ^18^F-FDG in each VOI are shown Figure 2. In the tumours, ^18^F-FAMT showed a shoulder peak immediately after the initial distribution (6 to 15 min and 2 min p.i., respectively), followed by gradual clearance, whereas in the granulomas, ^18^F-FAMT showed slow, exponential clearance after the initial distribution (2 min; SUV: 1.69 ± 0.25). The dynamic pattern of ^18^F-FAMT uptake in the tumours was significantly different from that in the granulomas or muscles (Figure 2a, *p* < 0.001). The dynamic pattern of ^18^F-FAMT uptake between granulomas and muscles showed no significant differences. The time-activity curves of ^18^F-FDG in the granulomas and tumours exhibited similar patterns and showed no significant differences (Figure 2b).

**Figure 3:**
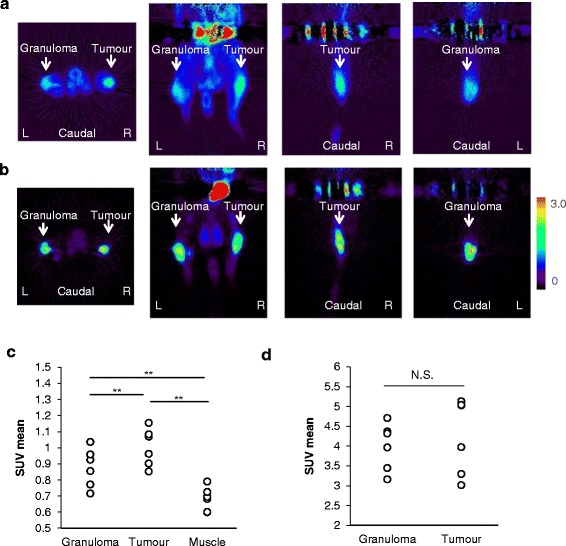
Static ^18^F-FAMT **(a)** and ^18^F-FDG **(b)** images in rats bearing granuloma and tumour. Transverse, coronal and sagittal images of ^18^F-FAMT and ^18^F-FDG (50 to 60 min). White arrow: locations of C6 tumour (right) and granuloma (left). Individual values of SUV for ^18^F-FAMT **(c)** and ^18^F-FDG **(d)** in granuloma and tumour lesion (50 to 60 min p.i.). Significant differences were determined (**p < 0.005; N.S., not statistically significant).

Static images of ^18^F-FAMT-PET and ^18^F-FDG-PET at 60 min after administration are shown in Figure 3a,b. Visual and SUV assessments of the static images were unable to differentiate tumours from granulomas in all cases, owing to overlaps in ^18^F-FAMT uptake, although the mean SUV in the granulomas (0.88 ± 0.12) was significantly lower than that in the tumours (1.00 ± 0.10, *p* < 0.001, Figure 3c). The static images and the mean SUVs of ^18^F-FDG in the granulomas were similar to those in the tumours (tumour vs. granuloma: 4.25 ± 0.93 vs. 4.00 ± 0.59, *p* = 0.37, respectively, Figure 3d).

**Figure 4:**
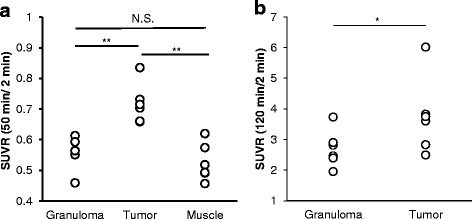
Individual values of SUVR for ^18^F-FAMT (50 min/2 min) **(a)** and ^18^F-FDG (120 min/2 min) **(b)**. Significant differences were determined (*p < 0.05; **p < 0.005; N.S., not statistically significant).

Representative SUVRs are shown in Figure 4. The SUVRs of ^18^F-FAMT in tumours were significantly higher than those in granulomas (50 min p.i./2 min p.i., tumour: 0.72 ± 0.06, granuloma: 0.56 ± 0.05, *p* < 0.001, Figure 4a), with no overlap between 20 and 100 min p.i.. The SUVRs in the granulomas and muscles showed no significant differences and had a considerable overlap owing to similarities in the dynamic profile. However, the SUVRs of ^18^F-FDG in tumours were relatively higher than those in granulomas (120 min p.i./2 min p.i., tumour: 3.57 ± 1.23, granuloma: 2.71 ± 0.60, *p* = 0.022, Figure 4b), although there was a substantial overlap.

### Immunofluorescent histochemistry

**Figure 5:**
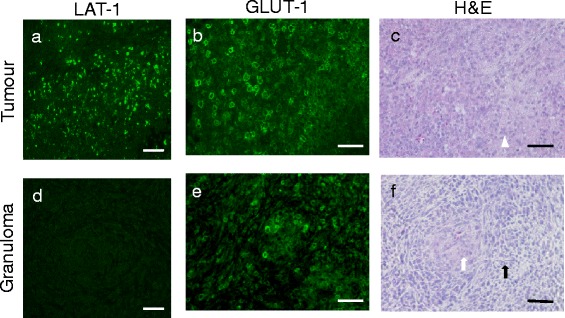
Microscopy images (×40) of immunofluorescent staining of LAT-1 (tumour **(a)**, granuloma **(d)**), GLUT-1 (tumour **(b)**, granuloma **(e)**) and H&E staining (tumour **(c)**, granuloma **(f)**). *White arrowhead*: cancer cell; *black arrow*: lymphocyte infiltration; *white arrow*: epithelioid cell granuloma. Bars indicate 50 μm.

High expression levels of LAT-1 were observed in the tumours (Figure 5a). In contrast, only faint expressions of LAT-1 were observed in the BCG-induced granulomas (Figure 5d). High expression levels of GLUT-1 were observed on cell membranes in both tumours and granulomas (Figure 5b,e). Representative images of H&E staining for the tumours and BCG-induced granulomas are shown in Figure 5c,f, respectively.

## Discussion

We found that dynamic ^18^F-FAMT PET is useful for differentiating granulomas from malignant tumours in a rat model. The moderate shoulder peak (6 to 15 min p.i.) and prolonged clearance in the time-activity curves of ^18^F-FAMT in tumours may reflect the high expression levels of LAT-1 in malignant lesions as compared to granulomatous lesions. The high expression levels of LAT-1 were confirmed by immunohistochemical staining. These results are consistent with previous clinical studies that showed a correlation between ^18^F-FAMT uptake and the expression levels of LAT-1 [16-18].

Although SUV assessment of ^18^F-FAMT showed significant differences between granulomas and tumours, the SUV value alone could not reliably provide the differential diagnosis in all cases, because the individual SUV values of tumours and granulomas overlapped substantially. It should be noted that unlike a clinical study of sarcoidosis patients [10], in our granuloma model, ^18^F-FAMT accumulated in the granulomas at 60 min p.i. Because the kinetics of ^18^F-FAMT depends on both increased local blood flow and LAT-1, one possible explanation for this difference is the effect of the concentration of radioactivity in the blood. However, even in the clinical study, the SUVs of ^18^F-FAMT in lung cancer lesions showed a substantial overlap with sarcoidosis lesions and background [10]. Because of the lower maximal SUVs, even in malignant lesions, the sensitivity of ^18^F-FAMT is lower than that of ^18^F-FDG [18,19]. Thus, a more definitive indicator that can improve the accuracy of the differential diagnosis is needed.

To address this issue, we evaluated the SUVR of ^18^F-FAMT relative to the SUV of 2 min p.i. and found that the overlap in SUVRs diminished and showed a significant difference in most of acquisition times (3 min p.i. to 120 min p.i.) because of prolonged retention of ^18^F-FAMT in the tumours. The SUVR appeared to depend on the existence of specific uptake, because there were no significant differences in the SUVRs between granulomas and muscle tissue where specific uptake was absent. These data suggested that the dynamic profile of ^18^F-FAMT in malignant lesions could reflect LAT-1-dependent specific uptake, regardless of the maximal SUV. Therefore, we believe that SUVR will be useful for differential diagnosis even if the maximal SUV is low. However, the calculation points should be optimized for each lesion, because differences between species or types of cancer might affect the absolute uptake or clearance rate of ^18^F-FAMT. Therefore, it is worth performing a clinical study to investigate whether SUVR measurement can improve the sensitivity of ^18^F-FAMT.

As predicted, ^18^F-FDG showed comparable time-activity curve patterns, SUVs and expression levels of GLUT-1 in tumours and granulomas. Although the SUVRs of ^18^F-FDG in the tumours were slightly higher than those in the granulomas, there was a considerable overlap. Therefore, ^18^F-FDG may provide less reliable information for the differential diagnosis, even if dynamic imaging is performed. These data support that BCG-induced granulomas could serve as a proper model for the differential diagnosis of tumours from inflammation, as was used in a study of ^11^C-MET [12].

However, unlike ^11^C-MET, which tended to be retained in both lesions after the initial distribution, ^18^F-FAMT showed slow, exponential clearance from both lesions. This difference might be attributed to the transport mechanism and metabolic pathway of the tracers. Indeed, ^11^C-MET can be a substrate for various transporters such as system L, A, ASC and y + L [20,21]. Additionally, following incorporation, ^11^C-MET is rapidly metabolized and trapped inside the cells [22]. These properties of ^11^C-MET may result in the retention of the tracer, both in tumours and in normal tissues. In contrast, ^18^F-FAMT is incorporated into cancer cells solely via LAT-1, which functions as a bi-directional transporter with an obligatory exchange mechanism [5,23]. The transport activity depends on the availability of intracellular exchange substrates with similar influx and efflux selectivity. Once incorporated, ^18^F-FAMT remained intact in cells for up to 60 min, and it was metabolized to protein to only a small extent [24]. These characteristics might cause a gentle decrease in ^18^F-FAMT in tumour cells along with a decrease in the concentration of ^18^F-FAMT in plasma. However, regardless of these properties, ^18^F-FAMT also showed distinct dynamic profiles in tumours and granulomas as ^11^C-MET did. Thus our results implicate potential wide applicability of dynamic PET for the differential diagnosis, even for the other amino acid tracers that show slow exponential clearance from the tumours.

Taken together, dynamic ^18^F-FAMT could be a more reliable imaging method for distinguishing tumours from granulomas, compared to conventional static images. Thus, dynamic ^18^F-FAMT PET in addition to standard ^18^F-FDG-PET would increase the accuracy of the differential diagnosis of malignant tumours from sarcoidosis, radiation-induced necrosis and tuberculosis. As dynamic ^18^F-FAMT showed comparable results to ^11^C-MET, clinical studies evaluating the applicability of dynamic amino acid PET for the differential diagnosis will be the objective of future research.

## Conclusions

The dynamic profile of ^18^F-FAMT showed significant differences between tumours and granulomas. Additionally, SUVR of ^18^F-FAMT diminished the overlap between tumours and granulomas, which has been observed in SUV analysis. These results indicate that dynamic ^18^F-FAMT and SUVR analysis might compensate for current imaging limitations and help with improving the diagnostic accuracy of ^18^F-FAMT.
